# Quantum-Dot-Induced Modification of Surface Functionalization for Active Applications of Whispering Gallery Mode Resonators

**DOI:** 10.3390/nano13131997

**Published:** 2023-07-03

**Authors:** Inga Brice, Vyacheslav V. Kim, Armands Ostrovskis, Arvids Sedulis, Toms Salgals, Sandis Spolitis, Vjaceslavs Bobrovs, Janis Alnis, Rashid A. Ganeev

**Affiliations:** 1 Laboratory of Quantum Optics, Institute of Atomic Physics and Spectroscopy, University of Latvia, Jelgavas 3, LV-1004 Riga, Latvia; inga.brice@lu.lv (I.B.); arvidssedulis@gmail.com (A.S.); janis.alnis@lu.lv (J.A.); 2 Laboratory of Nonlinear Optics, Institute of Astronomy, University of Latvia, Jelgavas 3, LV-1004 Riga, Latvia; vyacheslav.kim@lu.lv; 3 Department of Physics and Chemistry, Chirchik State Pedagogical University, 104 Amir Temur, Chirchik 111700, Uzbekistan; 4 Institute of Fundamental and Applied Research, TIIAME National Research University, 39 Kori Niyoziy, Tashkent 100000, Uzbekistan; 5 Institute of Telecommunications, Riga Technical University, Azenes 12, LV-1048 Riga, Latvia; armands.ostrovskis@rtu.lv (A.O.); toms.salgals@rtu.lv (T.S.); sandis.spolitis@rtu.lv (S.S.); vjaceslavs.bobrovs@rtu.lv (V.B.)

**Keywords:** whispering gallery mode resonator (WGMR), surface functionalization, quantum dots, optical frequency comb, third-harmonic generation

## Abstract

Quantum dots can modify the properties of the whispering gallery mode resonators (WGMRs) used in various potential applications. A deposition of a suitable nanomaterial for the surface functionalization of WGMRs allows for the achievement of high quality (Q) factors. Here, we show that the WGMR surface can be functionalized using quantum dots. We demonstrate that WGMRs covered with thin layers of HgS and PbS quantum dots are suitable for third-harmonic generation due to the high Q factor of the developed microresonators, thus significantly lowering the pumping power required for nonlinear optical interactions.

## 1. Introduction

In recent years, whispering gallery mode resonators (WGMRs) have attracted interest due to their various potential passive (filters [1], resonators [2], sensors [3,4]) and active (lasers [5], four-wave mixing [6]) applications. By choosing an appropriate material with very low absorption and fabricating a very smooth surface, WGMRs can reach ultra-high quality (Q) factors. Ultra-high Q allows the light circulating inside the WGMR to have a long-term interaction with the surrounding environment, thus enhancing the light–matter interaction. The key factors here are the application of suitable surrounding materials and WGMR geometry. The surface of WGMRs can be functionalized using different nanomaterials, which can significantly modify the optical properties of such resonators, which can be used, for instance, for the detection of specific molecules.

Microspheres represent the simplest geometry of WGMRs. Silica microspheres can be easily fabricated by melting the tip of an optical fiber. It is easy to coat such microspheres to functionalize the surface, which allows for the enhancement of their characteristics or the addition of new properties. Several coating methods are suitable depending on the geometry of such microresonators. For microsphere-based WGMRs, dip-coating or drop-casting methods are feasible.

We have previously coated WGMRs with different functionalizing layers. Gold nanoparticles [7] were used to enhance the sensitivity towards glucose oxidase to ensure the selectivity of this molecule [4]. WGMRs can be used to generate optical frequency combs (OFCs), which have many applications in optical clocks, spectroscopy, and communications. Silica microspheres have been used to generate an optical OFC inside a WGMR [6,8]. Overall, functionalization opens vast possibilities to tailor WGMRs to a wide range of sensor and biosensor applications.

One can assume that WGM resonators, especially those coated with nanostructures, are suitable for nonlinear optical interactions due to their ultra-high Q factors, thus significantly lowering the required pumping power for these processes. Recently, we have been interested in functionalizing the WGMR surfaces for active applications. Some initial tests were performed by coating WGMRs with nanoparticles and quantum dots (QDs). Notice that these nanostructures demonstrate a large nonlinear optical response [9]. In this paper, we prepared WGM microsphere resonators by melting a telecommunication fiber into a sphere. These WGMRs were functionalized with PbS and HgS QDs. During these studies, third-harmonic generation (THG) was observed using HgS quantum dots.

## 2. Materials and Methods

### 2.1. Fabrication of Microsphere Resonators

The most basic geometry of the WGM resonator is a microsphere. The fabrication of microspheres using melting is one of the fastest, cheapest, and most widely used manufacturing techniques. The tip of an optical fiber or silica glass rod is melted using heating by a gas flame [4], CO_2_ laser [10], or an arc discharge [11], thus allowing the surface tension of the liquid glass to create a sphere. Material absorption losses for silica are very low and limit the Q in the order of 10^11^ [12]. Water absorption by the silica surface, on the other hand, limits Q to below 10^9^ at 1550 nm [12].

WGM microsphere resonators can be fabricated by using the commercial arc fusion splicer. The first step of fabrication of the microspheres begins with the stripping and cleaning of a standard ITU-T G.652 compliant single-mode optical fiber for telecommunication purposes with core and cladding diameters of d = 8.2 µm and D = 125 µm, respectively. The second step of the fabrication process is placing the fiber inside the arc fusion splicer (Fujikura FSM-50S) and turning on the manual mode. The melting process requires multiple splicing cycles. The first couple of arc discharges of the fusion splicer melted the tip of the fiber and formed a sphere of about 170–190 µm. The diameter of the fiber limits the final size of the microsphere. Typically, the size will be between one and two times the diameter [13]. Additional discharges could be used to slowly increase the diameter of the microsphere, which is capped at around 300 µm. 

### 2.2. Characterization of Microresonators

The parameter often used to describe microresonators is the Q factor. The Q factor is a dimensionless quantity that describes the ability to store energy inside and determines the interaction length of light with the optical resonator. Several types of losses limit the maximum value of the optical Q factor in materials: surface scattering, material losses, the influence of the coupler (for example, coupling prism or tapered fiber), radiative loss, etc. [14]. Surface scattering is induced by imperfections or defects on the WGMR surface. Surface roughness can be the result of the capillary waves of the melted silica microsphere resonator. The surface might be “dirty” as tiny particles such as dust attach and are usually present on the surface. As we functionalize the surface of the microsphere resonators by coating them with nanostructures, the surface scattering loss mechanism is an important limiter for the Q factor.

Mathematically, the *Q* factor can be described as the product of the photon lifetime *τ* inside the cavity for a given resonant frequency *f* or the ratio of the resonant frequency to the spectral full width at half maximum (FWHM) ∆*f_FWHM_* of the resonance [15]:

(1) $$Q=2\pi f\tau =\frac{f}{\Delta f_{FWHM}}.$$

### 2.3. Surface Functionalization

The dip-coating method was used to prepare the microsphere samples coated with mercury sulfide (HgS) QDs and lead sulfide (PbS) QDs. This method is suitable for coating curved surfaces and is therefore usable for functionalizing the WGM microsphere resonators. One disadvantage of this method is the non-uniformity of the coating.

The application of nanoparticles instead of single particles (atoms and ions) can enhance the nonlinear optical response of the medium, in particular the nonlinear refraction, nonlinear absorption, etc. Additionally, the nonlinear optical features of nanoparticles and QDs have attracted great attention due to their applications in high-order harmonic generation. A relatively strong enhancement of high-order harmonic yield (up to 20×) was achieved in the laser-induced plasmas containing spherical nanoparticles of gold targets ablated in a vacuum using picosecond heating pulses [16]. It was shown that the efficient generation of harmonics can also be achieved in the plasmas containing QDs of semiconductor materials with regards to the plasmas containing atoms and ions of the same elemental state.

The details of the synthesis of HgS and PbS QDs were described elsewhere [17,18]. The initial concentrations of the quantum dot colloidal solutions were approximately similar (≈4 × 10^−5^ M). The mean size of the HgS QDs was 4 nm, and the mean size of the PbS QDs was 2.5 nm. Before dip coating, both colloidal solutions were diluted with ethanol. Dilution was necessary to reduce the number of QDs attaching to the surface of resonators, thus reducing the resulting scattering losses and rapid degradation of the Q factor. The PbS quantum dot solution was diluted at a 1:100 ratio, and the HgS quantum dot solution was diluted at a 1:10,000 ratio. The level of dilution was optimized for these two solutions by determining the minimal scattering losses and the smallest degradation of the Q factor. As HgS QDs were larger than PbS quantum dots, the former solution had to be diluted at a larger ratio. The WGM microsphere resonator was dipped in the diluted solution. The dipping speed was set to 0.2 mm/s. For samples coated multiple times, we waited at least 50 s to allow the solvent to evaporate before repeating the dipping.

### 2.4. Setup of the WGMR Testing System

**Figure 1:**
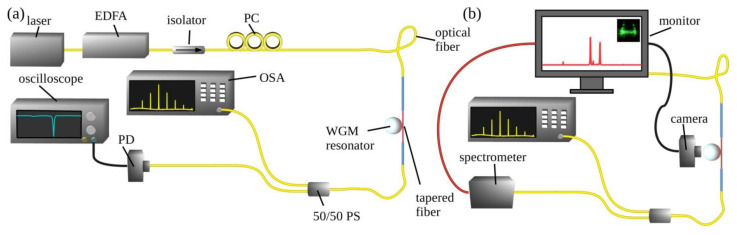
The basic scheme of the measurement test setups. (**a**) Test system setup with tapered fiber for 200 µm microsphere characterization and OFC detection. (**b**) Part of the system was modified for THG detection. EDFA: erbium-doped fiber amplifier, OSA: optical spectral analyzer, PC: polarization controller, PD: photodiode, PS: optical power splitter.

A basic scheme of the WGMR testing system is presented in Figure 1. For functionalized microresonator characterization fabricated by melting standard ITU-T G.652 single-mode telecommunication optical fiber with the fusion splicer, a tapered fiber coupling measurement system setup was used (Figure 1a). 

A pump laser was used for characterization at *λ* = 1550 nm and a linewidth of 50 kHz (SFL1550S, Thorlabs, Newton, USA) Another tunable laser (1465–1575 nm, Agilent 81989 A Tunable Laser, Bridge Tronic Global, Fountain Valley, USA), which could change the pumping frequency in steps in the telecom C-band range, was used for active applications. When necessary, the signal was amplified with an erbium-doped fiber amplifier (EDFA, Keopsys, Lannion, France). The isolator on the output was used to prevent the back-scattered light. The polarization state was adjusted using the polarization controller (PC) before coupling the optical signal into the microsphere through a tapered fiber. Two microscopes with zoom cameras were used to monitor the coupling position of the WGMR from the top and side views.

An optical power splitter (PS, splitting ratio 50/50) was used to monitor the transmission signal. The first output of the PS was connected to a InGaAs switchable gain amplified photo-detector (*λ* = 800–1700 nm) that was linked to an oscilloscope. This allowed us to record the transmission spectra when using a Thorlabs laser for microsphere characterization. The second output of the PS was connected to a high-resolution optical spectral analyzer (OSA, Q8384, Advantest Corporation, Tokyo, Japan, resolution 0.01 nm, or FTB-500, EXFO, Quebec, Canada, resolution 0.07 nm). This allowed us to record the OFC generation or lasing when using the Agilent 81989 A Tunable Laser (Bridge Tronic Global, Fountain Valley, CA, USA). The kick method was used to trigger the generation of the OFC or lasing instead of laser scanning [19]. The laser wavelength was changed using the step controls to generate the kick. The change of wavelength modulated the power of the pump laser for a short time. The power drop caused the resonator to cool rapidly and resonances to blue shift and thermally lock afterward [19].

Figure 1b shows the modifications made to the system for the detection of the THG signal in the case of the Agilent laser. The PS was connected to the OSA to monitor the OFC generation signal and spectrometer (Ocean Optics USB2000, resolution 0.5 nm) to monitor the THG spectrum simultaneously.

## 3. Results

**Figure 2:**
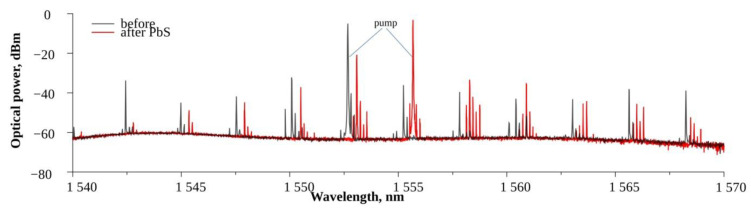
Generated optical frequency comb in 207 µm microsphere before (black line) and after (red line) functionalization with PbS quantum dots.

Functionalized WGM microsphere resonators were used to generate OFCs. The Q factor of the resonator before functionalization was 10^8^. A microsphere (diameter 207 µm) was functionalized with PbS quantum dots. Before functionalization, this microsphere generated an OFC with a free spectral range (FSR) of 350 ± 2 GHz appended with stimulated Brillouin scattering (SBS) peaks ≈20 GHz around each comb tooth line (see Figure 2, black line) when pumped with 1552.65 nm light. Brillouin scattering is a phenomenon that occurs due to the nonlinearity of a medium when light interacts with the acoustic phonons of the material. SBS occurs when the intensity of the pumping light is high enough. After functionalization, the microsphere generated a similar OFC with the same FSR of 350 GHz when pumped with 1555.67 nm light (Figure 2, red line). Moreover, the SBS comb lines were even more pronounced. 

**Figure 3:**
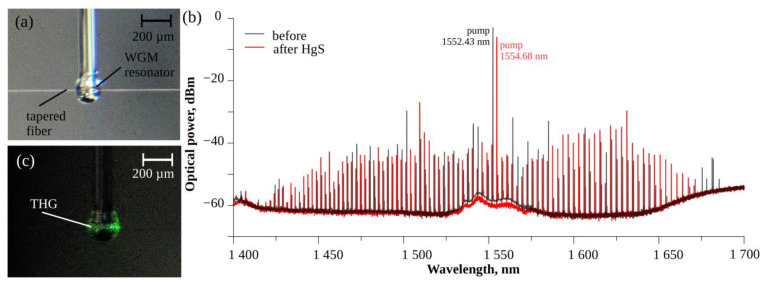
Microsphere functionalized with HgS quantum dots. (**a**) 183 µm microsphere sample coupled using tapered fiber. (**b**) Generated optical frequency comb before (black line) and after (red line) functionalization with HgS quantum dots. (**c**) Third-harmonic generation.

A microsphere (diameter 183 µm, Figure 3a) was functionalized with HgS quantum dots. Before functionalization, this microsphere generated an OFC with an FSR of 362 GHz (Figure 3b black line) when pumped with 1552.43 nm light. After functionalization, the microsphere generated a similar OFC with the same FSR of 362 GHz when pumped with 1554.68 nm light (Figure 3b red line). Comparing the registered OFC pump intensities, after the deposition of HgS, the intensity dropped by ~2.8 dB, suggesting that more of the pump’s power is used for the OFC and THG processes. The interesting feature of these studies is the observation of the third-harmonic generation for the WGM resonator samples coated with HgS QDs (Figure 3c). 

**Figure 4:**
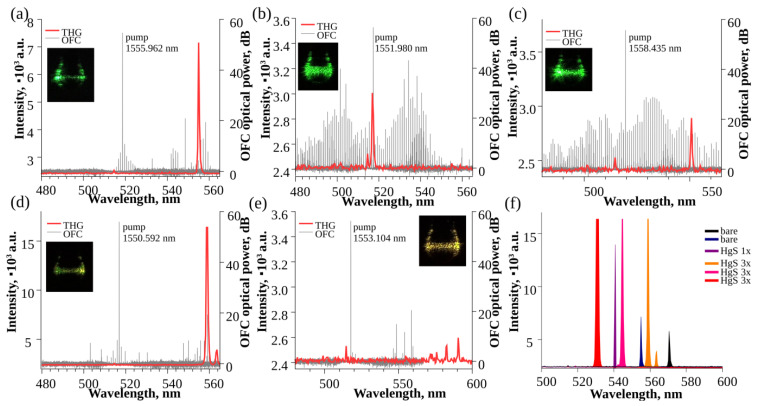
Third-harmonic generation for second WGM microsphere functionalized with HgS quantum dots: THG detected with a spectrometer (red line) compared with the registered OFC spectra displayed with wavelength divided by three (gray line) (**a**) before functionalization when pumped with 1555.962 nm light and after functionalization dip-coated in HgS QD solution three times when pumped with (**b**) 1551.980 nm, (**c**) 1558.435 nm, (**d**) 1550.592 nm, and (**e**) 1553.104 nm light; (**f**) THG signal intensity comparison for before and after functionalization one and three times with HgS quantum dots. Insets show visual THG detected with a camera.

The second WGM microsphere (diameter of 185 µm) was dip-coated in HgS QD solution three times. The Q factor after functionalization was 2.8 × 10^7^. When pumping with 1556.769 nm, the simultaneous generation of a 358 GHz OFC and continuous THG was recorded. Figure 4 shows the comparison of the third-harmonic generation spectra (red line) recorded using a spectrometer with the OFC generated inside the WGM microsphere (gray line). 

To compare both signals, the OFC spectrum wavelength was divided by three to match the THG wavelength. The wide arrays of generated colors from green to red depending on the wavelength of the pump laser were observed. The green THG was excited by the laser pump or one of the OFC lines near the pump both before (Figure 4a) and after functionalization (Figure 4b,c). In Figure 4b, the pumping laser at 1551.980 nm generated the THG signal at 516 nm. A weaker THG signal at 514 nm could have been generated by other OFC lines. Similarly, in Figure 4c, other lines of the main OFC pumped with 1558.435 nm emission generated the THG signal at 514 nm and 551 nm. A green-yellow third-harmonic generation was produced by the Raman OFC lines pumped with 1550.592 nm emission and generated around 1650–1700 nm (Figure 4d). The comparison of THG and OFC for the yellow-orange-red region is not possible due to the spectral range of the used OSA. However, we believe that the THG was produced by the OFC lines generated outside the detection range (Figure 4e). 

In Figure 4f, the most intense recorded THG signals are compared before functionalization (black and dark blue lines), after functionalization by dip coating once in HgS quantum dot solution (purple line), and after functionalization by dip coating the resonator three times in HgS quantum dot solution (red, magenta, and orange lines). After functionalization, the THG signal intensity significantly increased. 

**Figure 5:**
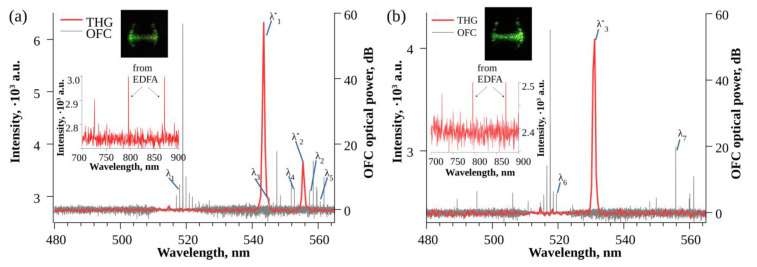
Sum-frequency generation through the cascaded second-harmonic generation and sum-frequency generation. THG detected with a spectrometer (red line) compared with the registered OFC spectra displayed with wavelength divided by three (gray line) (**a**) before functionalization when pumped with 1556.718 nm light and (**b**) after functionalization dip-coated in HgS quantum dot solution three times when pumped with 1552.695 nm light. The insets show the spectra in the region of SHG.

Some registered THG spectra did not have any obvious OFC lines in the comparison region (Figure 5). Instead of direct THG from the OFC line c/λ* = 3c/λ, the harmonic can be generated either through cascaded second-harmonic generation (SHG) from one OFC line and sum-frequency generation (SFG) of a second harmonic and a different OFC line [20] or from the third-order sum-frequency generation (TSFG), which can be based on the third-order combinations of two OFC lines [21]. 

For a stronger peak at λ_1_* = 543.5 nm in Figure 5a, a signal in the vicinity of the third harmonic can be generated from the OFC line λ_1_ = 1553.86 nm coupled with two photons of line 1672.18 nm: c/λ_1_* = c/λ_1_ + 2c/λ_2_. However, to realize such a process (SHG + OFC), the second-harmonic signal from the λ_2_ pump should be detected. In the meantime, no strong spectral lines corresponding to the second-harmonic emission of the λ_2_ pump were observed (see insets to Figure 5a). Because of this, TSFG can be considered as a rather suitable mechanism for the observed spectra. To prove these assumptions, we analyzed the observed generated spectra.

A very weak spectral line at around 836 nm was registered. For the stronger peak at λ_2_* = 555.4 nm shown in Figure 5a, the sum-frequency signal can be generated either through c/λ_2_* = c/λ_3_ + 2c/λ_5_ or c/λ_2_* = 2c/λ_4_ + c/λ_5_, where λ_3_ = 1635.49 nm, λ_4_ = 1658.41 nm, and λ_5_ = 1681.96 nm. Again, very weak spectral lines around 824 nm and 840 nm were registered (Figure 5a, inset). For the peak at λ_3_* = 531.1 nm shown in Figure 5b, the sum-frequency signal can be generated from the two photons of the OFC line λ_6_ = 1558.58 nm together with the photon from the OFC line λ_7_ = 1667.45 nm: c/λ_3_* = 2c/λ_6_ + 2c/λ_7_. We observed a weak spectral line at 781 nm (Figure 5b, inset). It is hard to expect that the SHG intensity could be sufficient for sum-frequency generation. Additionally, the SHG signal had a lower coupling efficiency back into the tapered fiber compared with the THG signal. This was observed when the red emission could be seen using a camera, but no intense signal was observed using a spectrometer.

It was possible to functionalize the WGM microsphere resonators with PbS and HgS QDs and preserve the generation of OFCs. The optical nonlinearity of the QDs is incorporated with the OFCs. In the case of PbS quantum dots, already-present SBS was enhanced, and in the case of HgS quantum dots, a generation of third harmonic from different OFC lines was detected. After functionalization with HgS quantum dots, it was easier to excite continuous THG when pumping in the 1550–1560 nm range. Additionally, THG intensity under these conditions was increased. Our studies showed that the QDs have the potential for active applications as a functionalization material for WGMRs. Further studies are necessary to clarify the mechanisms allowing THG in the case of HgS QD-coated WGM resonators, while restricting similar processes in PbS quantum dots. 

## 4. Discussion

Over the last decades, *χ*^(3)^-assisted third-harmonic generation processes were studied theoretically and realized experimentally in various types of microcavities, including microdroplets, silica toroid/microsphere/microbottle, SiN microring, silicon photonic-crystal micro-/nanocavities and microcavities with hybrid materials [9,22,23,24,25,26,27,28,29,30]. Our studies demonstrate further amendments to microresonator functionalization using QDs, confirm the above assumptions, and show the perspectives of QDs applications in higher-order harmonics generation through the analysis of their frequency-generation properties.

There is not much work reported on the application of deposited nanoparticles for the functionalization of microresonators, as one can judge taking into account the analysis reported in [9]. Moreover, to the best of our knowledge, there are no reports on the use of QDs for these purposes. In [9], the authors demonstrated a ~3-fold enhancement in third-harmonic generation conversion efficiency using indium tin oxide nanoparticles on the surface of an ultra-high-Q silica microsphere compared with a pure silica microsphere. In our case, we registered a weak THG in the case of the pure silica microsphere; however, it was challenging to obtain a continuous THG signal, which was observed in the case of the HgS coating. It is difficult to determine the enhancement factor of such a coating in our case, but it was larger than the one achieved in the abovementioned reference. 

As it was mentioned, there are two explanations for the observed THG spectra other than a direct third-harmonic generation from the OFC line (c/λ^∗^ = 3c/λ). The first one is the mechanism described in [20] as a sum-frequency generation of the weak second harmonic and one of the OFC lines. The second one [21] analyzes a variety of *χ*^(3)^ nonlinear interactions in microspheres, consisting of THG and Raman-assisted third-order sum-frequency generation in the visible range. In the most general case of third-order sum-frequency generation, three different waves interact with a nonlinear medium to generate a fourth wave of different frequency (ω_TSFG_ = ω_1_ + ω_2_ + ω_3_). If the three input frequencies degenerate, it will result in THG. In this case, the energy conservation requires ω_THG_ = 3ω_1_. Thus, to realize this mechanism of THG, one has to have a strong Raman emission. Apart from that, those studies were carried out using an erbium-doped microresonator. 

Notice that we tried to observe THG using microresonators coated with erbium and did observe the green emission in our experimental conditions. However, the signal did not couple back into the tapered fiber and could not be recorded at the time. We did not include those studies since the topics of our research were the applications of QDs for the modulation of the properties of microresonators. Meanwhile, the sum-frequency generation in the case of the microresonators coated with HgS QDs was stable and relatively strong. These observations pointed out the large probability of the Raman-assisted third-order sum-frequency generation in our case. 

We observed a second-harmonic emission in the 778 nm region. It was expectedly weak. Meanwhile, this weak second harmonic originated from the silica glass rather than from the QD coating. Additionally, the second-harmonic signal did not couple back into the tapered fiber as efficiently as a third-harmonic signal. When the red emission (778 nm) was observed with the camera, the signal detected with the spectrometer was very weak.

The presence of HgS in the form of QDs, where all mercury sulfide molecules are combined in a statistically homogeneous spatial distribution, points out the almost centrosymmetric features of this structure. This significantly diminishes the SHG conversion efficiency. All QDs can be considered as almost centrosymmetric species, thus notably diminishing the probability of the even harmonics generation, especially the higher-order even harmonics. 

The relatively high conversion efficiency towards the third harmonic of the 900–1700 nm 150 fs pulses in the thin (70 nm) film containing HsS QDs deposited on the glass substrates has earlier been reported in [17]. The intensity, polarization, and spectral dependencies of this process in HgS QD thin film have been analyzed. The third-harmonic conversion efficiency was measured to be 7 × 10^−4^. Notice that second-harmonic generation was not reported in those studies. 

PbS QDs can also be considered as almost centrosymmetric species. Their low-order nonlinear optical properties were analyzed in [18]. Though those studies have shown that the larger PbS QDs (9 nm) demonstrate stronger nonlinear optical effects, such as nonlinear refraction and nonlinear absorption, compared with the smaller QDs (2.5 nm), no THG was reported in the case of those two groups of QDs. Similarly, no low-order harmonics were observed in the present study in which microresonators were coated with thin layers of PbS QDs. 

## 5. Conclusions

The surface of the WGMR was functionalized with quantum dots to enhance its optical properties. The functionalization of microresonators was explored by finding the optimal conditions for the deposition of thin layers of HgS and PbS quantum dots. The coated WGMRs preserved the OFC generation and allowed a lower-order harmonic generation. We demonstrated that WGMRs covered with a thin layer of quantum dots are suitable for third-harmonic generation due to their ultra-high Q factors, thus significantly lowering the pumping power required for the nonlinear optical interactions. It was shown that some THG spectra were produced through the cascaded second-harmonic generation and sum-frequency generation.

## Data Availability

The data that support the findings of this study are available on request from the corresponding author.
